# Heterogeneous cognitive and neuroimaging profiles in older adults with type 2 diabetes: the modifying effect of coronary artery disease

**DOI:** 10.3389/fnagi.2026.1825819

**Published:** 2026-07-15

**Authors:** Qingjuan Ren, Lei Zhi, Hongfang Liu

**Affiliations:** Department of Geriatrics, Shijiazhuang People’s Hospital, Shijiazhuang, China

**Keywords:** brain MRI, cognitive impairment, coronary artery disease, older adults, type 2 diabetes mellitus

## Abstract

**Background:**

Cognitive impairment in older adults with type 2 diabetes mellitus (T2DM) is increasingly recognized, and coronary artery disease (CAD) is also linked to cognitive decline. However, their combined impact remains underexplored. This exploratory study characterized neurocognitive profiles and brain MRI features associated with comorbid CAD in older T2DM patients with cognitive impairment.

**Methods:**

This cross-sectional study screened 1,217 older adults with T2DM; 523 with cognitive impairment (MoCA < 26) were included. All underwent brain MRI and were stratified by CAD status. Between-group comparisons were performed to examine differences in cognitive and neuroimaging measures, with false discovery rate correction applied for multiple comparisons. Multiple linear regression was used to assess the independent association between CAD and cognitive function. Logistic regression was further performed to evaluate the association between CAD and binary neuroimaging outcomes. Finally, two-step cluster analysis was performed to identify distinct cognitive and brain imaging profiles among these participants.

**Results:**

Patients with T2DM and CAD exhibited higher frequencies of cerebrovascular markers: lacunar infarcts (74.65% vs. 57.10%, *p* < 0.001), strategic infarcts (32.86% vs. 19.35%, *p* = 0.001), and white matter hyperintensities (66.67% vs. 36.77%, *p* < 0.001), but lower medial temporal lobe atrophy (30.52% vs. 44.52%, *p* = 0.001). After false discovery rate correction, these differences remained significant. The CAD group scored lower on MoCA total, visuospatial/executive function, attention, and orientation. CAD independently predicted lower global cognition (*B* = −0.74, 95% CI: −1.42 to −0.06, *p* = 0.03). Logistic regression further confirmed that CAD was independently associated with higher odds of lacunar infarcts, strategic infarcts, and white matter hyperintensities (all *p* < 0.05), but not with medial temporal lobe atrophy (*p* = 0.518). Cluster analysis revealed distinct profiles: among non-CAD patients, three subgroups (cerebrovascular-predominant, mixed, atrophy-predominant); among CAD patients, two subgroups (both with significant lacunar infarcts, differentiated by atrophy prominence and delayed recall).

**Conclusion:**

In older T2DM patients with cognitive impairment, comorbid CAD was independently associated with a vascular-dominant phenotype. Cluster analysis suggested CAD may modify phenotypic heterogeneity, though these exploratory findings require validation. They provide a hypothesis-generating framework for integrating cardiovascular risk management into cognitive treatment.

## Introduction

Cognitive impairment is a prevalent and disabling condition among older adults, imposing a substantial burden on both individuals and healthcare systems (Gracner et al., 2025). The two most common forms are Alzheimer’s disease (AD), characterized by progressive neurodegeneration and predominantly medial temporal lobe atrophy on neuroimaging, and vascular dementia, which results from cerebrovascular injury and typically presents with white matter hyperintensities, lacunar infarcts, and other ischemic lesions (Gan et al., 2024). Type 2 diabetes mellitus (T2DM) and coronary artery disease (CAD) have each been independently associated with an increased risk of cognitive decline. Notably, patients with T2DM have a significantly elevated risk of mild cognitive impairment, with estimates reaching as high as 60%, and a 50–100% increase in dementia risk compared to non-diabetic populations. Prediabetes has also been linked to the progression from cognitive impairment to dementia (Srikanth et al., 2020). Similarly, patients with CAD have a 27% increased risk of dementia (Wolters et al., 2018). Although previous research has separately examined the impact of T2DM or CAD on cognitive function, the combined effect of these two conditions in older adults remains insufficiently explored. Moreover, there is a lack of comprehensive analysis regarding the pattern of impairment across specific cognitive domains, such as memory, executive function, attention, and information processing speed, in this comorbid context.

The present study therefore aimed to clarify the neurocognitive and structural neuroimaging profiles of older adults with T2DM and comorbid CAD. By identifying distinct cognitive and brain MRI characteristics in this population, we seek to illuminate the underlying pathophysiology of cognitive decline in this comorbid population and inform the development of targeted management strategies.

## Methods

### Research design

This cross-sectional study enrolled older adults with T2DM who underwent cognitive assessment using the Montreal Cognitive Assessment (MoCA). Individuals with cognitive impairment were identified as the study population. Baseline demographic and clinical information was collected through structured questionnaires, after which all participants underwent standardized 3.0T brain MRI.

The study was conducted in accordance with the Declaration of Helsinki and was approved by the Ethics Committee of Shijiazhuang People’s Hospital (Approval No: 2025138). All participants provided written informed consent.

### Participants

Between January 2024 and September 2025, a total of 1,217 older adults with T2DM underwent cognitive screening at our hospital. Of these, 523 individuals met the criteria for cognitive impairment and were included in the final analysis, including 281 males and 242 females.

The inclusion criteria were as follows: (1) age ≥ 65 years, with a clinical diagnosis of T2DM (based on WHO 2023 criteria) and cognitive impairment (MoCA score < 26); (2) adequate communication and cognitive ability to complete the assessments; and (3) willingness and ability to provide written informed consent.

Patients were excluded if they met any of the following criteria: (1) a history of acute cerebrovascular events within the past 6 months (e.g., stroke); (2) contraindications to MRI; (3) active malignant tumor; or (4) incomplete data collection that compromised the evaluation of primary outcomes.

### Data collection and definitions

#### Baseline clinical assessment

Baseline clinical information was systematically collected, covering participant demographics, educational attainment, and detailed medical history.

T2DM was diagnosed based on the American Diabetes Association criteria: fasting plasma glucose ≥7.0 mmol/L; 2-h plasma glucose ≥11.1 mmol/L during an oral glucose tolerance test; glycated hemoglobin (HbA1c) ≥ 6.5%; or current use of glucose-lowering medications. Diabetes duration was calculated from the date of first diagnosis to study enrollment.

CAD was defined based on documented medical history and clinical evidence, including any of the following: (1) a history of myocardial infarction; (2) prior coronary revascularization (percutaneous coronary intervention or coronary artery bypass grafting); (3) angiographically or coronary CT angiography confirmed coronary stenosis ≥50% in at least one major epicardial artery; (4) a documented diagnosis of CAD by a physician accompanied by consistent antianginal medication use (e.g., nitrates, beta-blockers) and/or objective evidence of myocardial ischemia (e.g., ischemic electrocardiographic changes, positive stress test). CAD status was ascertained through comprehensive review of medical records, including hospitalization notes, cardiac imaging reports, and medication prescriptions.

The medical history also comprised prior cerebrovascular events (including cerebral infarction and cerebral hemorrhage).

#### Brain MRI acquisition

All participants underwent brain MRI using a 3.0T MRI scanner (Siemens 3.0T; Lumina) according to a standardized brain imaging protocol. The imaging sequences included T1-weighted imaging (T1WI), T2-weighted imaging (T2WI), fluid-attenuated inversion recovery (FLAIR), and diffusion-weighted imaging (DWI). All MRI images were independently evaluated by two neuroradiologists who were blinded to all clinical information. Inter-rater agreement was assessed using kappa statistics, and any discrepancies were resolved through consensus.

The imaging features were systematically categorized and scored as follows:

#### Cerebrovascular measures

##### Lacunar infarcts

Lacunar infarcts were defined as round or ovoid subcortical lesions, 3–15 mm in diameter, exhibiting hyperintensity on T2WI/FLAIR and hypointensity on T1WI (Regenhardt et al., 2018).

##### Strategic infarcts

Strategic infarcts were defined as infarcts occurring in brain regions critically involved in cognitive networks, where even focal damage may result in cognitive impairment. The assessed strategic regions encompassed the left frontotemporal areas, left thalamus, right parietal lobe, and hippocampus. Participants were classified as having a strategic infarct if radiologically confirmed infarcts (acute or chronic) were identified in any of these regions (Weaver et al., 2021).

##### White matter hyperintensities

White matter hyperintensities (WMH) were rated on T2WI/FLAIR images using the Fazekas scale, which separately evaluates periventricular WMH (PVWMH) and deep WMH (DWMH), each scored from 0 to 3 (Wahlund et al., 2001). The global WMH burden was expressed as the sum of the two sub-scores (range 0–6). For analytical purposes, a Fazekas total score ≥2 was defined as clinically significant WMH, consistent with established clinical classification thresholds where scores of 1–2 represent mild, 3–4 moderate, and 5–6 severe white matter disease.

#### Atrophy measures

##### Medial temporal lobe atrophy

Medial temporal lobe atrophy (MTA) was assessed on coronal T1WI using the standardized MTA visual rating scale (score 0–4 per hemisphere) (Wahlund et al., 2017). The more severely affected side was used for analysis. In accordance with age-adjusted cut-offs, an MTA score ≥ 2 was considered abnormal for participants younger than 75 years, while a score ≥ 3 was defined as significant atrophy for those aged 75 years or older.

##### Global cerebral atrophy

Global cerebral atrophy was evaluated on axial FLAIR images using the Global Cortical Atrophy (GCA) scale, ranging from 0 (no atrophy) to 3 (severe diffuse cortical atrophy with “knife-blade” gyral appearance). A GCA score ≥ 2 was defined as clinically significant global atrophy (Wahlund et al., 2017).

### Assessment of cognitive function

Global cognition was assessed using the MoCA scale, which evaluates eight aspects of cognitive function: visuospatial/executive function, naming, memory, attention, language fluency, abstraction, delayed recall, and orientation. The total score ranges from 0 to 30, with higher scores indicating better cognitive performance. To adjust for the influence of educational attainment, one point was added to the total score for participants with 12 or fewer years of formal education; however, the total score was capped at 30. A MoCA score of < 26 was considered indicative of cognitive impairment (Nasreddine et al., 2005).

### Statistical analysis

All statistical analyses were performed using SPSS version 26. Continuous variables are presented as mean ± standard deviation, while categorical variables are summarized as frequencies and percentages. Differences between groups for continuous variables were assessed using Student’s t-test, and differences in categorical variables were evaluated using the chi-square test. To control for multiple comparisons, the Benjamini-Hochberg false discovery rate (FDR) correction was applied separately to the neuroimaging features (5 comparisons) and the cognitive domain scores (8 comparisons). Adjusted q-values < 0.05 were considered statistically significant.

Multiple linear regression was employed to analyze the association between CAD and cognitive performance. To further examine whether MTA provides additional explanatory power for global cognition beyond GCA, a hierarchical linear regression was performed with MoCA total score as the dependent variable. To explore the neuroimaging characteristics and cognitive profiles between the CAD and non-CAD groups, a two-step cluster analysis was applied to MRI derived features and cognitive domain scores. Results of the clustering were visualized using a heatmap, in which continuous data were standardized by Z-scores and categorical data were represented by standardized residuals.

A two-tailed *p*-value of < 0.05 was considered statistically significant for all analyses.

## Results

### Characteristics of T2DM patients with CAD: neuroimaging and cognitive patterns

The baseline and clinical characteristics of the study participants are summarized in Table 1. Among the 523 participants with cognitive impairment, 213 (40.73%) had comorbid CAD. After FDR correction for multiple comparisons, the significant differences in neuroimaging and cognitive measures remained significant. As shown, T2DM patients with CAD had a higher prevalence of lacunar infarcts (74.65% vs. 57.10%, *p* < 0.001, *q* < 0.001), strategic infarcts (32.86% vs. 19.35%, *p* = 0.001, *q* = 0.003), and WMH (66.67% vs. 36.77%, *p* < 0.001, *q* < 0.001), while showing a lower prevalence of MTA (30.52% vs. 44.52%, *p* = 0.001, *q* = 0.003). Cognitively, the CAD group had significantly lower scores on the MoCA total score, visuospatial/executive function, and attention than the non-CAD group (all *q* < 0.001).

**Table 1:** Comparison of baseline characteristics, brain MRI features, and cognitive function between T2DM patients with and without CAD.

Characteristic	CAD group (*n* = 213)	Non-CAD group (*n* = 310)	*p*-value	*q* (FDR)
Demographics				
Age, years	74.41 ± 4.79	73.71 ± 5.12	0.115	—
Education, years	6.90 ± 2.63	6.62 ± 2.62	0.228	—
Male, *n* (%)	115 (54.00)	166 (53.54)	0.929	—
Neuroimaging features				
Lacunar infarcts, *n* (%)	159 (74.65)	177 (57.10)	<0.001	<0.001
Strategic infarcts, *n* (%)	70 (32.86)	60 (19.35)	0.001	0.002
WMH (Fazekas≥2), *n* (%)	142 (66.67)	114 (36.77)	<0.001	<0.001
Medial temporal lobe atrophy, *n* (%)	65 (30.52)	138 (44.52)	0.001	0.001
Global cerebral atrophy (GCA ≥ 2), *n* (%)	41 (19.25)	81 (26.13)	0.074	0.074
Cognitive function (MoCA domains)				
MoCA total score	20.94 ± 3.61	22.13 ± 3.67	<0.001	<0.001
Visuospatial/executive function	3.46 ± 0.94	3.92 ± 1.08	<0.001	<0.001
Naming	2.61 ± 0.94	2.62 ± 0.50	0.542	0.542
Attention	3.66 ± 1.10	4.35 ± 1.28	0.000	<0.001
Language fluency	2.42 ± 0.71	2.41 ± 0.65	0.862	0.862
Abstraction	1.49 ± 0.66	1.54 ± 0.65	0.356	0.406
Delayed recall	2.67 ± 1.39	2.84 ± 1.18	0.154	0.206
Orientation	4.63 ± 1.27	4.40 ± 1.05	0.031	0.062

Type 2 diabetes mellitus, T2DM; coronary artery disease, CAD; magnetic resonance imaging, MRI; White matter hyperintensities, WMH; montreal cognitive assessment, MoCA.

### Association between CAD and cognitive function

Multiple linear regression analysis was conducted with the MoCA total score as the dependent variable. The model included CAD status as the primary independent variable, while adjusting for age, sex, education duration, and diabetes duration.

**Figure 1:**
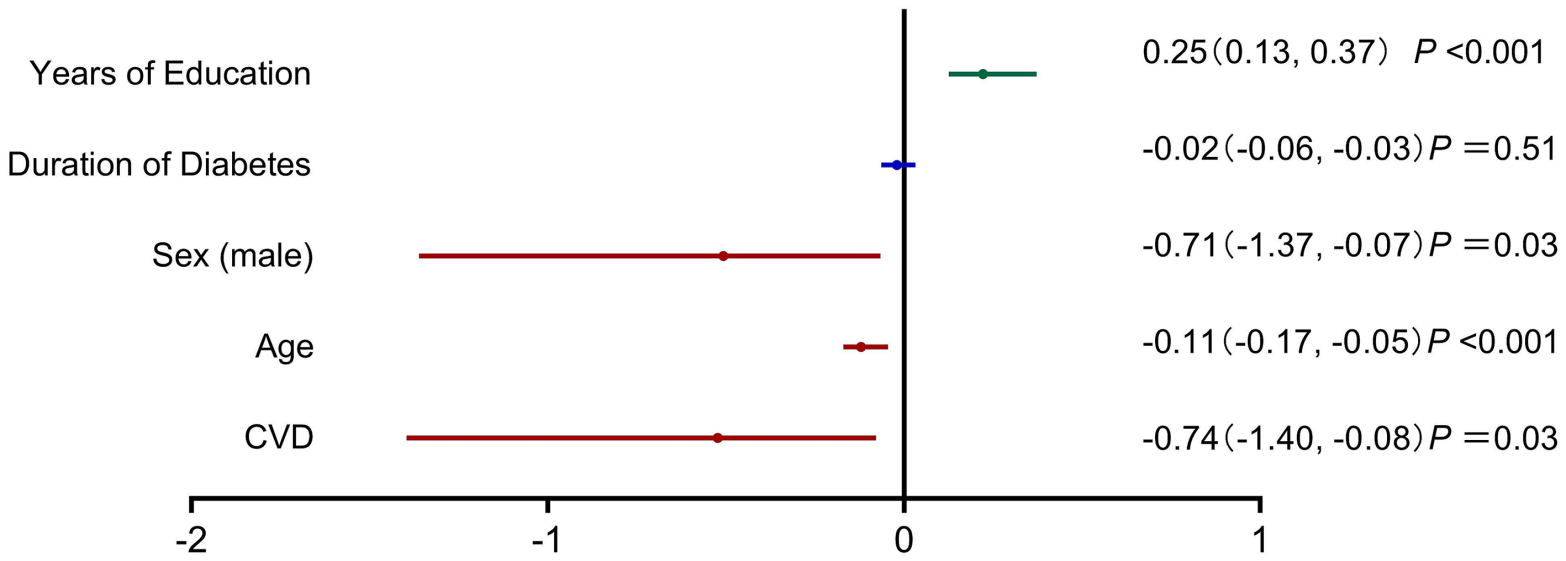
Factors associated with MoCA scores in older adults with T2DM. Forest plot showing standardized beta coefficients from multiple linear regression analysis with MoCA score as outcome. CAD presence (*B* = −0.74, *p* = 0.03), older age (*B* = −0.11, *p* < 0.001), and male sex (B = −0.71, *p* = 0.03) were negatively associated with cognition; education duration showed a positive association (*B* = 0.25, *p* < 0.001). Diabetes duration was non-significant (*p* = 0.51).

As shown in Figure 1, the presence of CAD was significantly associated with lower MoCA scores (*B* = −0.74, *p* = 0.03). Older age (*B* = −0.11, *p* < 0.001) and male sex (*B* = −0.71, *p* = 0.03) were also independently associated with poorer cognitive performance, whereas longer education duration was positively associated with MoCA scores (*B* = 0.25, *p* < 0.001). No significant association was observed between diabetes duration and MoCA scores (*B* = −0.02, *p* = 0.51).

### Association between CAD and neuroimaging outcomes

After adjusting for age, sex, education, and diabetes duration, CAD remained significantly associated with lacunar infarcts (OR = 1.456, *p* = 0.037), strategic infarcts (OR = 1.718, *p* = 0.003), and white matter hyperintensities (OR = 1.540, *p* = 0.018). No significant associations were found for medial temporal lobe atrophy (OR = 0.888, *p* = 0.518) or global cerebral atrophy (OR = 1.438, *p* = 0.083). Full results are presented in Supplementary Table S1.

### Association between MTA and cognitive function adjusted for GCA

Given that the non-CAD group had a higher prevalence of MTA than the CAD group (Table 1), we further examined whether MTA provides additional explanatory power for global cognition beyond GCA. Hierarchical regression was performed with MoCA total score as the dependent variable. Model 1 included GCA, age, sex, education years, and diabetes duration, which significantly explained 5.3% of the variance (*R^2^* = 0.053, *p* < 0.001). Adding MTA in Model 2 did not significantly improve the model (Δ*R^2^* = 0.002, *p* = 0.286), indicating that MTA does not independently contribute to global cognitive performance after accounting for GCA.

### Cluster analysis of neurocognitive subtypes by CAD status

**Figure 2:**
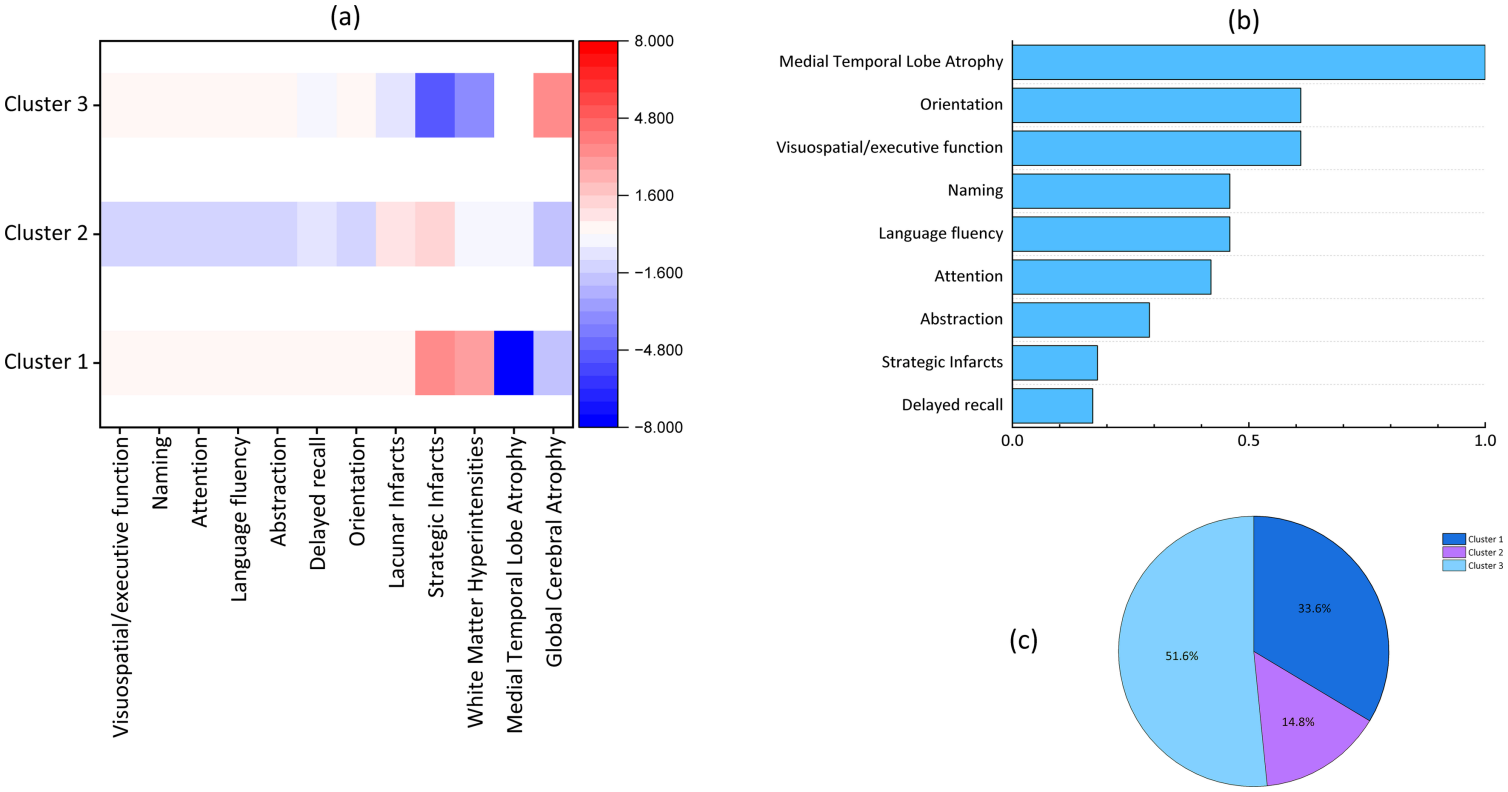
cluster analysis of brain imaging and cognitive characteristics in patients without coronary artery disease. Two-step cluster analysis (SPSS 26) was performed using cerebrovascular markers (lacunar infarcts, strategic infarcts, white matter hyperintensities), atrophy markers (medial temporal lobe atrophy, global cerebral atrophy), and MoCA domain scores. **(a)** heatmap of feature patterns across clusters **(b)** ranked feature importance for cluster discrimination **(c)** distribution of patient clusters.

Two-step cluster analysis was performed separately for patients with and without CAD to identify distinct profiles based on neuroimaging features (cerebrovascular markers: lacunar infarcts, strategic infarcts, WMH; atrophy markers: MTA, GCA) and cognitive performance. In patients without CAD (Figure 2), three distinct subgroups were identified, accounting for 33.6, 14.8, and 51.6% of the cohort, respectively. Cluster 1 (33.6%) showed a higher frequency of strategic infarcts and WMH, along with a lower frequency of brain atrophy measures, and exhibited cognitive performance close to the sample average across most domains. Cluster 2 (14.8%) exhibited features of both lacunar and strategic infarcts alongside brain atrophy, while also demonstrating the lowest cognitive scores across all assessed domains. Cluster 3 (51.6%) exhibited a higher frequency of MTA and GCA, coupled with a lower frequency of cerebrovascular lesions, and showed near-average cognitive performance except for slightly lower scores in delayed recall.

**Figure 3:**
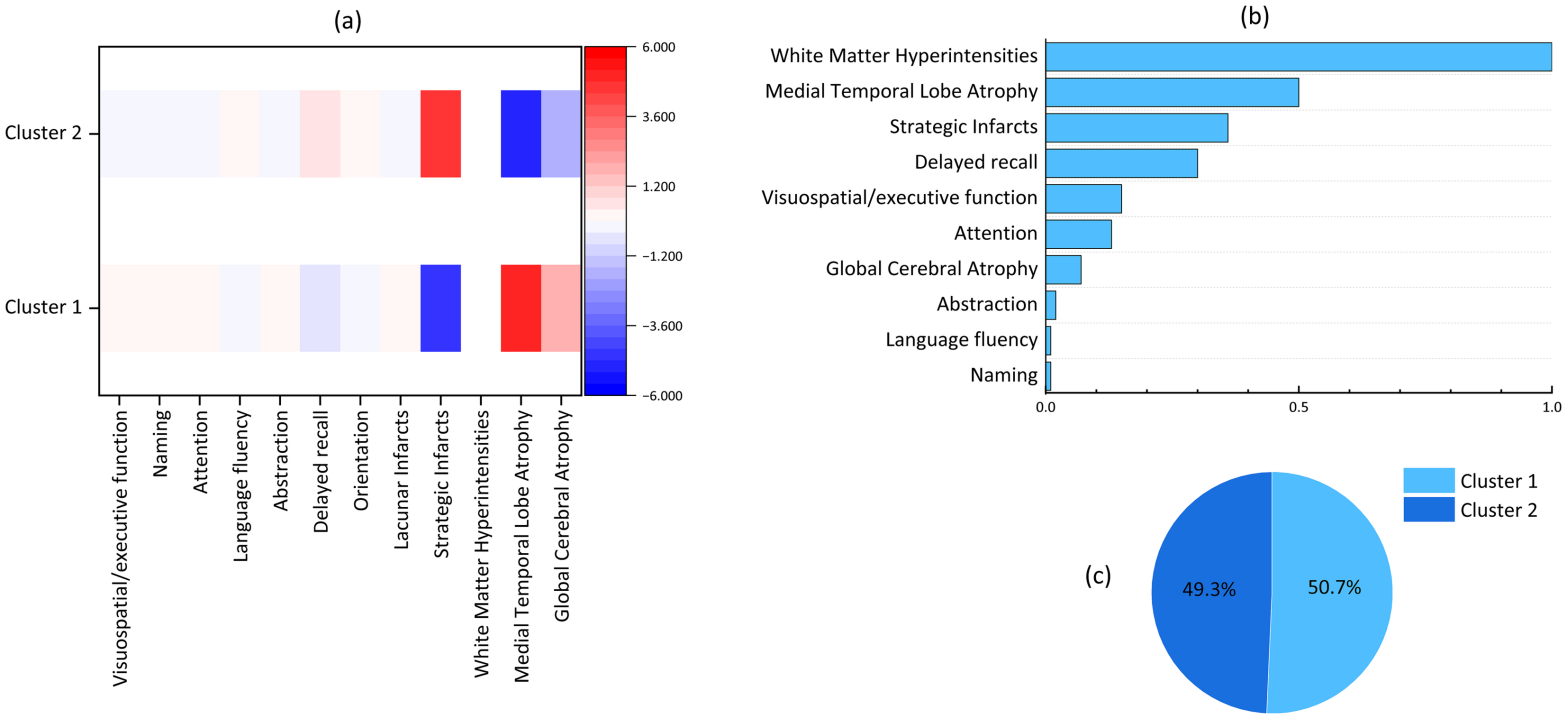
Cluster analysis of brain imaging and cognitive characteristics in patients with coronary artery disease. The same clustering procedure and variables as in Figure 2 were used. **(a)** heatmap of feature patterns across clusters **(b)** ranked feature importance for cluster discrimination **(c)** distribution of patient clusters.

In patients with CAD (Figure 3), two distinct subgroups were identified. Cluster 1 (50.7%) showed a higher frequency of MTA and GCA, along with a lower frequency of strategic infarcts and WMH, and demonstrated slightly lower scores in delayed recall. Cluster 2 (49.3%) demonstrated a higher frequency of strategic infarcts and WMH, together with a lower frequency of atrophy measures, and exhibited cognitive performance close to the sample average across most domains, with slightly higher scores in delayed recall.

## Discussion

### CAD as an independent risk factor for cognitive impairment in older adults with T2DM

This study investigated the impact of CAD on cognitive function in older adults with T2DM. The CAD group showed a significantly lower total MoCA score compared with the non-CAD group. Multivariate linear regression analysis, after adjusting for confounders including age, sex, education duration, and diabetes duration, confirmed that CAD is an independent risk factor for cognitive impairment. Cognitive impairment, a common clinical syndrome in the older population, arises primarily from vascular factors and neurodegenerative pathology. These two pathological processes often coexist and interact synergistically, creating a cumulative effect that accelerates cognitive aging (Godrich et al., 2022). T2DM significantly increases the risk of cognitive decline through multiple interrelated pathophysiological pathways (Peng et al., 2024; Biessels and Despa, 2018): First, cerebral insulin resistance serves as a key mechanism. Brain regions critical for memory, such as the hippocampus and cortex, contain abundant insulin receptors. The development of cerebral insulin resistance promotes Aβ deposition and Tau protein hyperphosphorylation, thereby contributing to the pathogenesis of AD. Second, a complex pathological network comprising oxidative stress with mitochondrial dysfunction, neuroinflammation, and vascular impairment collectively exacerbates neuronal damage, disrupts cerebral blood supply, and accelerates cognitive deterioration.

The distinctive contribution of this study lies in revealing that comorbid CAD further elevates the risk of cognitive impairment in T2DM beyond the established mechanisms. Neuroimaging results indicated a significantly higher prevalence of lacunar infarcts, strategic infarcts, and white matter hyperintensities in the CAD group compared to the non-CAD group, suggesting that cerebrovascular pathology may represent an important mechanism through which CAD exacerbates cognitive decline. Both CAD and cerebral infarction, as common macrovascular complications of diabetes, share atherosclerosis as their fundamental pathological basis. This common etiology underlies their strong tendency for comorbidity, further amplifying the risk of cognitive impairment in this vulnerable population (Sattar et al., 2023). We wish to clarify that the lower prevalence of MTA in the CAD group is not unexpected and should not be interpreted as a protective effect of CAD. Rather, it reflects competing etiologies: in T2DM patients with CAD, cognitive impairment is predominantly driven by vascular pathology, so the proportion of patients with prominent hippocampal atrophy is naturally lower. Notably, the sensitivity analysis showed that MTA did not independently predict global cognition beyond GCA. This likely reflects the strong correlation between regional and global atrophy, as well as the relative insensitivity of the MoCA total score to mild memory deficits.

### CAD exerts a “pruning effect” on cognitive phenotypes in T2DM

Current research has confirmed that CAD is closely associated with cognitive decline, and its link to Vascular dementia is significantly stronger than that to Alzheimer’s disease (Liang et al., 2023; Olesen et al., 2025). In the non-CAD group of the present study, 51.6% of patients predominantly exhibited brain atrophy on neuroimaging, 33.6% showed a predominance of vascular lesions, and 14.8% presented with both imaging features simultaneously; this latter group had the poorest cognitive performance. In the CAD group, CAD may exert a potential “pruning effect” on cognitive phenotypes. The patterns of cognitive impairment converge into two exploratory patterns: one predominantly characterized by atrophy dominance (50.7%), and the other by vascular injury dominance (49.3%). This suggests that in the context of T2DM, systemic vascular pathology, represented by CAD, may reshape or even mask other neuropathological phenotypes through its dominant pathological processes, thereby highlighting and reinforcing the crucial role of vascular mechanisms in cognitive impairment. The underlying mechanisms may involve the following four aspects: (1) Cerebral hypoperfusion and energy metabolic crisis. Reduced cardiac output and impaired vascular regulation resulting from CAD can lead to chronic cerebral hypoperfusion, causing insufficient energy supply to neurons and increasing their susceptibility to ischemic injury. In patients with concomitant atrial fibrillation, the risk of recurrent ischemic stroke is further elevated. Under such pathological conditions, even if Alzheimer’s disease-related pathology is present, its clinical manifestations may be “masked” by more prominent vascular cognitive impairment (AlRawili et al., 2025; Xu et al., 2021). (2) Exacerbation of systemic inflammatory responses. CAD, especially when complicated by heart failure, constitutes a state of chronic low-grade inflammation. Emerging evidence shows that in diabetic CAD patients, elevated levels of the advanced glycation end product Nε-carboxymethyl-lysine (CML) are significantly correlated with increased IL-6 and TNF-*α*, as well as decreased nitric oxide, indicating that the AGE-RAGE axis may drive both systemic inflammation and endothelial dysfunction (Eiras, 2024). The release of inflammatory factors such as IL-6, TNF-α, and CRP can disrupt the blood–brain barrier or activate cerebral vascular endothelial cells, amplifying intracerebral inflammation and vascular injury, thereby serving as a key driver of cerebrovascular pathology (AlRawili et al., 2025; Anderle et al., 2025). (3) Shared pathological basis between CAD and vascular cognitive impairment (VCI). CAD and VCI, particularly cerebral small vessel disease (CSVD), share multiple risk factors, including age, hyperglycemia, hypertension, and dyslipidemia. These conditions demonstrate substantial overlap in their pathophysiological mechanisms. CSVD is widely recognized as the most common pathological substrate for VCI and may contribute to more than half of dementia cases. Characteristic MRI findings encompass lacunar infarcts, WMH, cerebral microbleeds, enlarged perivascular spaces, and in advanced stages, cerebral atrophy. These structural changes are associated with cognitive deficits, most notably slowed information processing speed (Markus and Joutel, 2025; Blevins et al., 2021). In this study, 49.3% of type 2 diabetes patients with cerebrovascular lesions demonstrated both imaging features and cognitive impairment patterns consistent with cerebrovascular pathology. This finding suggests that in this population, cerebrovascular disease and cognitive impairment may represent distinct tissue-level manifestations of the same systemic disorder. (4) Potential shared genetic susceptibility between CAD and VCI. Evidence indicates that CAD and VCI may share certain genetic predispositions. Some gene variants appear to simultaneously elevate the risk of both cardiovascular diseases and cognitive decline, implying that individuals genetically susceptible to CAD may also face an increased risk of developing dementia in later life (Sittichokkananon et al., 2025). Furthermore, cognitive impairment can reduce treatment adherence, health literacy, and self-management abilities, leading to inadequate cardiovascular treatment, thereby creating a mutually reinforcing cycle between the two conditions (Jamil et al., 2025). This understanding holds significant implications for elucidating the mechanistic role of comorbidities in cognitive impairment, suggesting that interventions targeting systemic inflammation and cerebral perfusion may provide unique neuroprotective benefits for T2DM patients with comorbid coronary artery disease.

### Clinical and research implications

These exploratory findings provide a preliminary basis for subtype-guided management of cognitive impairment in T2DM, pending external validation. For the cerebrovascular-predominant subtype, strict vascular risk factor control is the primary strategy. For the atrophy-predominant subtype, neurodegenerative-directed approaches (e.g., memory-targeted therapies) may be considered. In patients with comorbid CAD, integrated cardiovascular-cognitive management is crucial, as CAD treatment may improve cognitive outcomes and vice versa (van Nieuwkerk et al., 2023). This calls for interdisciplinary collaboration among endocrinology, cardiology, and neurology to shift from single-disease care to holistic patient-centered management.

## Limitations and future directions

This study has several limitations. First, its retrospective cross-sectional design precludes causal inference and the observation of dynamic subtype evolution. Second, we lacked Alzheimer’s disease-specific biomarkers (e.g., Aβ-PET) and a CAD-only group without T2DM, limiting our ability to confirm neuropathological mechanisms and to determine whether the observed patterns are specific to the T2DM population. Third, our analysis was restricted to T2DM patients with established cognitive impairment, which limits generalizability to the broader T2DM population and may introduce selection bias (e.g., referral patterns for cognitive testing or MRI). Fourth, data on key confounders (e.g., hypertension, HbA1c, prior stroke, other vascular comorbidities, and medication use) were unavailable, so residual confounding cannot be ruled out; therefore, our findings should be interpreted as associations rather than causal effects. Future prospective studies with comprehensive covariate assessment and biomarker collection are needed to validate our findings and refine subtype-specific intervention strategies.

## Conclusion

In older T2DM patients with cognitive impairment, comorbid CAD was independently associated with a vascular-dominant phenotype. Cluster analysis suggested CAD may modify phenotypic heterogeneity, though these exploratory findings require validation. They provide a hypothesis-generating framework for integrating cardiovascular risk management into cognitive treatment.

## Data Availability

The raw data supporting the conclusions of this article will be made available by the authors, without undue reservation.
