# Supplementary material for: Heterogeneous cognitive and neuroimaging profiles in older adults with type 2 diabetes: the modifying effect of coronary artery disease
Source: Front Aging Neurosci. 2026 Jul 15;18:1825819. doi: 10.3389/fnagi.2026.1825819 (PMC13416259; doi:10.3389/fnagi.2026.1825819)
Supplement: Supplementary file 1 [file Table_1.DOCX]

Supplementary Table 1. Association between CAD and neuroimaging outcomes

| Neuroimaging outcome | OR (95% CI) | ***p*-value** |
| --- | --- | --- |
| Lacunar infarcts | 1.456 (1.023–2.073) | 0.037 |
| Strategic infarcts | 1.718 (1.201–2.457) | 0.003 |
| White matter hyperintensities | 1.540 (1.077–2.201) | 0.018 |
| Medial temporal lobe atrophy | 0.888 (0.620–1.273) | 0.518 |
| Global cerebral atrophy | 1.438 (0.954–2.166) | 0.083 |

Note: Logistic regression, adjusted for age, sex, education, and diabetes duration.

Abbreviations: CAD, coronary artery disease; OR, odds ratio; CI, confidence interval.
